# Methodology used to develop the minimum common data elements for surveillance and Reporting of Musculoskeletal Injuries in the MILitary (ROMMIL) statement

**DOI:** 10.12688/f1000research.152514.2

**Published:** 2025-04-03

**Authors:** Garrett S. Bullock, Joanne L. Fallowfield, Sarah J. de la Motte, Nigel Arden, Ben Fisher, Adam Dooley, Neil Forrest, John J. Fraser, Alysia Gourlay, Ben R. Hando, Katherine Harrison, Debra Hayhurst, Joseph M. Molloy, Phillip M. Newman, Eric Robitaille, Deydre S. Teyhen, Jeffrey M. Tiede, Emma Williams, Sandra Williams, Damien Van Tiggelen, Joshua J. Van Wyngaarden, Richard B. Westrick, Carolyn A. Emery, Gary S. Collins, Daniel I. Rhon

**Affiliations:** 1 Centre for Sport, Exercise, and Osteoarthritis, Nuffield Department of Orthopaedics, Rheumatology and Musculoskeletal Sciences, University of Oxford, Oxford, England, UK; 2 Department of Biostatistics and Data Science, Wake Forest University School of Medicine, Winston-Salem, North Carolina, USA; 3 Department of Orthopaedic Surgery & Rehabilitation, Wake Forest University School of Medicine, Winston-Salem, North Carolina, USA; 4 Institute of Naval Medicine, Directorate of People and Training, Royal Navy, Hampshire, UK; 5 Consortium for Health and Military Performance, Department of Military and Emergency Medicine, F. Edward Hébert School of Medicine, Uniformed Services University, Bethesda, MD, USA; 6 Department of Physical Medicine & Rehabilitation, F. Edward Hébert School of Medicine, Uniformed Services University, Bethesda, Maryland, USA; 7 Medical Research Council (MRC), Environmental Epidemiology Unit, University of Southampton, Southampton, England, UK; 8 Defence Primary Healthcare, Headquarters Surgeon General, London, UK; 9 Army Health, Army Headquarters, London, UK; 10 Human Sciences Programme, Defence Science & Technology, New Zealand Defence Force, Auckland, New Zealand; 11 Operational Readiness and Health Directorate, Naval Health Research Center, San Diego, CA, USA; 12 Joint Health Command, Department of Defence, Australian Capital Territory, Australia; 13 Army-Baylor University, Waco, Texas, USA; 14 Department of Rehabilitation Medicine, Brooke Army Medical Center, Fort Sam Houston, Texas, USA; 15 Defense Statistics Health, Ministry of Defence, London, UK; 16 Director General, Finance, Ministry of Defence, London, UK; 17 Headquarters Defence Medical Services, Strategic Command, Ministry of Defence, London, UK; 18 Formerly at the Physical Performance Service Line, Office of the Army Surgeon General, Falls Church, Virginia, USA; 19 Research Institute for Sport and Exercise, University of Canberra, Canberra, Australian Capital Territory, Australia; 20 Teaching Stream Department of Physical Therapy, Temerty Faculty of Medicine, University of Toronto, Toronto, Ontario, Canada; 21 31 CF H Svcs C Detachment, Department of National Defence, Meaford, Canada; 22 Defense Health Network, National Capital Region, Defense Health Agency, Bethesda, MD, USA; 23 Second Health Brigade, Australian Army, Sydney, Australia; 24 Defence Rehabilitation, Ministry of Defence, London, UK; 25 Belgium Armed Forces, Brussels, Belgium; 26 59th Medical Wing, Joint Base San Antonio - Wilford Hall Ambulatory Surgical Center, Lackland AFB, Texas, USA; 27 Military Performance Division, US Army Research Institute of Environmental Medicine, Natick, Massachusetts, USA; 28 Sport Injury Prevention Research Centre, Faculty of Kinesiology, University of Calgary, Calgary, Alberta, Canada; 29 Centre for Statistics in Medicine, Nuffield Department of Orthopaedics, Rheumatology and Musculoskeletal Sciences, University of Oxford, Oxford, England, UK

**Keywords:** Implementation Science, Consensus, Delphi, Military, Sports Medicine, Military Medicine, Wounds and Injuries, Investigative Techniques

## Abstract

**Background:**

The objective was to summarize the methodology used to develop the international minimum data elements for surveillance and Reporting of Musculoskeletal Injuries in the MILitary (ROMMIL) statement. This is a recommended list of elements to be collected and reported when conducting injury surveillance research in military settings.

**Methods:**

A Delphi methodology was employed to reach consensus. Preliminary steps included conducting a literature review and surveying a convenience sample of military stakeholders to 1) identify barriers and facilitators of military musculoskeletal injury (MSKI) prevention programs, 2) identify relevant knowledge gaps, and 3) establish future research priorities. A sequential three-round Delphi consensus survey followed, including relevant stakeholders from militaries around the world, using results to conduct an asynchronous knowledge user meeting (mixture of in-person and live video conference and recording) to explore the level of agreement among subject matter experts. Knowledge users, including former and current military service members, civilian practitioners working in military health networks, and international subject matter experts having experience with policy, execution, or clinical investigation of MSKI mitigation programs, MSKI diagnoses, and MSKI risk factors in military settings. For each round, participants scored questions on a Likert scale of 1-5. Scores ranged from No Importance (1) to Strong Importance (5).

**Results:**

Literature review and surveys helped inform the scope of potential variables. Three rounds were necessary to reach minimum consensus. Ninety-five, 65, and 42 respondents participated in the first, second and third rounds, respectively.

**Conclusions:**

Achieving consensus across relevant knowledge users representing military organizations globally can be challenging. This paper details the methodology employed to reach consensus for a core minimum data elements checklist for conducting MSKI research in military settings and improve data harmonization and scalability efforts. These methods can be used as a resource to assist in future consensus endeavors of similar nature.

## Introduction

Collection, surveillance, and reporting of injury data is an important and ubiquitous aspect of musculoskeletal (MSK) care within systems seeking to evaluate and improve injury prevention and care models. This is particularly true across military performance and medical settings. ^1^^–^^3^ Often the data are inadequately powered (individual clinics or small sample sizes), requiring merging of data to properly compare patient subgroups, generalize results across different populations, and potentially answer clinically important questions. ^4^^,^^5^ However, merging of these data is often hampered by high heterogeneity in data elements, outcomes, and definitions. ^1^ Thus, there have been many recommendations to standardize data collection elements and practices to improve harmonization of data within a specific discipline or setting. ^5^^–^^7^

Data collection procedures are highly variable across and even within military organizations world-wide, without a universal standard operating procedure. ^1^ For example, in a systematic review assessing musculoskeletal injury (MSKI) risk factors in military populations, high variability in exposure, outcome, and predictor collection and reporting from 170 studies limited the ability to effectively compare risk factors and injury risk across service member groups. ^1^ In a scoping review of 132 articles investigating military MSKI mitigation programs, heterogeneity in data and outcomes hindered the ability to calculate service member injury burden. ^8^ This variability in the collection and reporting of data elements may prevent clinicians, military leaders, and researchers from answering pertinent operational and clinical questions.

One method to improve consistency of standardized data collection and reporting across studies is through the development of a recommended list of core minimum data elements that everyone should collect and report. ^5^^,^^7^ Consensus for core minimum data elements has occurred across biomedicine fields (e.g. geriatrics, ^7^ pediatrics ^5^) and for specific conditions (e.g. traumatic injuries, ^9^ osteoarthritis, ^10^ pain ^6^). A common list of standardized data elements enables a systematic approach to data collection and focused analysis, allowing the specific study question to be addressed, but also facilitating aggregation and meta-analysis across studies. ^4^ This standardized practice ultimately leads to greater potential for increased cohort size and ultimately inference power. ^4^

Recently a group of knowledge users (person who contributed to knowledge creation and have input equal to the scientific community) consisting of clinicians, researchers, policymakers and leaders in military settings world-wide collaborated to identify/recommend the minimum elements for collection and reporting in any study assessing MSKI in military populations. The project followed a Delphi consensus methodology to engage many relevant stakeholders world-wide. This paper summarizes the details and methodology used to develop the Minimum Data Elements for surveillance and Reporting of Musculoskeletal Injuries in the MILitary (ROMMIL) statement.

## Methods

### Process

**Figure 1.**
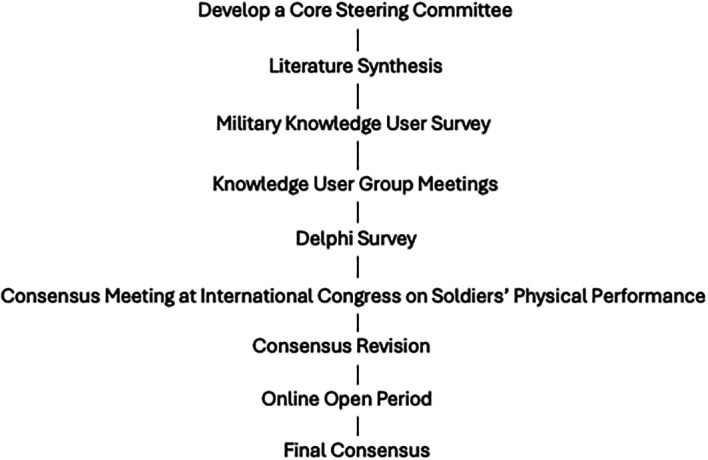
Consensus process.

The ROMMIL project was informed by the guidance for developers of health research guidelines. ^11^ Consensus reporting was informed by the ACcurate COnsensus Reporting Document (ACCORD) reporting guidelines. ^12^ Further methods and details, and respective protocols are available on Open Science Framework ( https://osf.io/2wqbr/). Figure 1 displays the consensus process. This project adhered to all ethical principles of the Declaration of Helsinki. It was determined to not be human subjects research by the Institutional Review Board at Wake Forest University, School of Medicine (# IRB00115873), and therefore formal consent was not necessary. All participants were made aware that the data and collected information would be used to formulate a consensus statement, with the intent to publish the results, and all participants were invited to participate in the full process as authors.

### Strategy

A core group of six members (GB, NA, JF, SdlM, BF, DR) identified the need for a standardized list of minimum core data elements for collection and reporting when conducting MSKI research. They pursued a balance in expertise across clinical and research disciplines, countries, and military branches while conducting a literature synthesis via scoping review, knowledge user survey, Delphi study and consensus meeting to achieve the ROMMIL consensus statement.

### Bias Reduction

Measures to reduce bias included having steering group members not being able participate in the small group conversations and only contribute to the full group discussions during the consensus meetings when invited. Steering group members were not allowed to initiate remarks. If invited to speak, steering group members were only allowed to provide clarification or guidance when appropriate. An independent experienced external moderator lead all group discussions to reduce the risk of dominant personalities and also reduce steering group bias. Finally, all meetings were open for in person, video, and viewing at a later date, with an open period to comment, to allow for inclusion of voices that could not make the meetings.

### Stage 1: Literature synthesis

A scoping review was performed to (1) identify barriers to and facilitators of military MSKI prevention programs, (2) identify relevant knowledge/information gaps and (3) establish future research priorities. This scoping review has been published separately. ^8^ Databases included MEDLINE and the Defense Technical Information Center (DTIC). The results from the scoping review were merged with recommendations provided by the entire author group. The group created a preliminary list of all possible data elements and principles, based on the results of the scoping review. A knowledge user meeting, consisting of 45 participants, was then convened to further evaluate the list, which then received final approval by the steering committee (GB, NA, JF, SdlM, BF, DR).

### Stage 2: Survey

The authors recruited a cross-sectional convenience sample the Total Force Fitness MSK Health conference attendees, then used a snowball recruitment method to gather knowledge user feedback concerning military MSKI mitigation program barriers and facilitators. The detailed results from this survey were reported elsewhere. ^13^ The reported barriers and facilitators were used to help guide the framework questions for the consensus exercise.

### Knowledge user involvement (Patient and public involvement)

Knowledge users (including former and current military service members, civil practitioners that work in military health networks, and international subject matter experts having experience with policy, execution, or clinical investigation of MSKI mitigation programs, MSKI diagnoses, and military MSKI risk factors) were included in the development of the research question. A working group evolved from a sample of the knowledge users after the initial meetings. Knowledge users sought to improve the overarching question while accounting for research implications and facilitate knowledge translations. The knowledge user group included active-duty service members, veterans, military leaders, medical professionals that work in military settings (both military and civilian, that include athletic trainers, physical therapists, and physicians), scientists that work in military settings, and exercise physiologists from the United Kingdom and United States.

### Delphi study

*Study design*

A sequential three round Delphi survey was performed. An asynchronous knowledge user meeting was performed to explore level of agreement among subject matter experts.

*Recruitment*

Experts were identified through the closeness continuum. ^13^ The closeness continuum identifies inclusive experts with subjective, mandated, and objective closeness to the topic of interest. ^13^^,^^14^ Military service members having sustained MSKI across their careers had subjective closeness. Clinicians treating service members for MSKI had mandated closeness. Researchers and scientific experts investigating military MSKI had objective closeness.

Recruitment entailed an email identifying an individual as a military MSKI expert and requesting that they participate in a Delphi consensus project, via a series of planned meetings. A follow-up email with an encrypted link was sent to those who wanted to participate, in order to answer a series of questions concerning injury surveillance and reporting in military settings (see extended data). ^15^ Participants were told that their answers would help inform the consensus project, and that all responses would remain anonymous.

*Delphi rounds and scoring*

Participants scored each question on a Likert scale of 1-5 for each round. Each question followed one of two formats shown in Table 1.

**Table 1.** Likert survey question format.

Please refer to the scale below to rate how important you think each data point is to include in military musculoskeletal injury research:
No importance
Minimal importance
Some importance
Strong importance
Extreme importance

Please refer to the scale below to rate how important you think each injury classification system or metric is to define military musculoskeletal injury:
Extremely inappropriate
Somewhat inappropriate
Neither appropriate nor inappropriate
Somewhat appropriate
Extremely appropriate

Following round 1, a synchronous knowledge user virtual meeting was held to discuss outlying scores, express and document disagreement or dissent concerning any statement, and propose alterations to variables and/or verbiage.

In round 2, participants had access to their previous scoring for each question. Changes in scoring were documented. A third round of scoring was unnecessary if the knowledge user group reached consensus on a given question during the second round.

Consensus criteria are defined *a priori* ( Table 2).

**Table 2.** Criteria for consensus for inclusion, consensus for exclusion, and non-consensus.

Criteria	Definition	Implication
Threshold for consensus that variable should be included in a minimum data set for musculoskeletal injury in military populations (Consensus for inclusion).	≥ 80% of participants assign a score of ≥ 4 OR Median score of ≥4 in two consecutive rounds.	Variable does not proceed to the next round.
Threshold for consensus that variable should not be included in a minimum data set for musculoskeletal injury in military populations (Consensus for exclusion).	≥ 50% of participants assign a score of ≤ 2 OR Median score of ≤2 in two consecutive rounds.	Variable does not proceed to the next round.
Threshold for non-consensus.	Median score of 3 in two consecutive rounds OR Median score decreases by ≥ 1 point in a round compared to the previous round.	Variable does not proceed to the next round, consensus for exclusion.
	Median score increases by ≥ 1 point in a round compared to the previous round.	Variable proceeds to the next round.

*Statistical analyses*

Missing data were assessed prior to analyses. A complete case analysis was performed for the Delphi study. Participant data were reported as mean (standard deviation) for continuous data and count (%) for nominal and ordinal data. All analyses were performed in R 4.2.1 (R Core Team (2023). R: A language and environment for statistical computing. R Foundation for Statistical Computing, Vienna, Austria. URL https://www.R-project.org/). The *dplyr* package was used for cleaning and analyses, and the *ggplot2* package was used for data visualization. The raw data available from each round are available in an open-source repository. ^16^

### Stage 3: Consensus meeting

After completing the Delphi rounds, a final consensus meeting was held on 13 September 2023 at the International Congress on Soldier’s Physical Performance, in London, United Kingdom. All participants were invited to attend. The meeting was recorded for people who could not join the meeting in person to view at a later date. The purpose of the meeting was to (1) discuss the literature synthesis, survey, and Delphi survey results and (2) obtain advisory input on the final set of recommended minimum data elements for MSKI research in military settings. Fifty international participants (including military MSKI knowledge users, clinicians, and research experts) were present. The meeting was facilitated by the ROMMIL steering committee (GB, NA, JF, SdlM, BF, DR). Participants discussed and sought to achieve consensus on each data element during the meeting.

### Stage 4: Consensus revision

After the consensus meeting, the steering committee (GB, NA, JF, SdlM, BF, DR) reviewed the results and revised the consensus statement based on pertinent feedback. The format and wording of each data element were reviewed and agreed upon, taking into consideration the survey, Delphi study, and consensus meeting results and discussions.

A 6-week open comment period was held for anyone to comment on the document. The electronic link was made available through the Musculoskeletal Injury Rehabilitation Research for Operational Readiness (MIRROR) website ( https://mirrorusuhs.org/) and emailed to individuals who had participated in preliminary meetings and activities.

The draft consensus was then circulated again among consensus participants to (1) confirm accurate representation of the group consensus or (2) determine if further clarification was needed.

## Results

Ninety-five, 65 and 42 respondents participated in the first, second and third rounds of the Delphi, respectively ( Table 3). The process used a Delphi methodology, which is an acceptable and preferred method for validating consensus experiments. ^16^^,^^17^

**Table 3.** Delphi participant descriptive statistics.

Variable	Round one participants (n = 95)	Round two participants (n = 65)	Round three participants (n = 45)
Primary Role			
Administrative Leadership	10 (11%)	4 (6%)	4 (9%)
Clinician/Practitioner	25 (26%)	23 (35%)	11 (24%)
Command/Leadership	5 (5%)	1 (2%)	3 (7%)
Implementing Human Performance Programs	4 (4%)	3 (5%)	4 (9%)
Implementing Injury Prevention Programs	9 (9%)	8 (12%)	3 (7%)
Researcher	41 (43%)	24 (37%)	20 (44%)
Secondary Role			
Administrative Leadership	11 (12%)	6 (9%)	3 (7%)
Clinician/Practitioner	23 (24%)	13 (20%)	12 (28%)
Command/Leadership	3 (3%)	2 (3%)	2 (4%)
Implementing Human Performance Programs	10 (11%)	9 (14%)	7 (16%)
Implementing Injury Prevention Programs	20 (21%)	18 (28%)	7 (16%)
Physical Training Instructor	3 (3%)	1 (2%)	0 (0%)
Researcher	13 (14%)	8 (12%)	7 (16%)
Country			
Australia	14 (15%)		
Belgium	2 (2%)		
Canada	7 (7%)		
New Zealand	2 (2%)		
United Kingdom	23 (24%)		
United States	47 (49%)		

* Blank cells denote information was not disclosed

### Round one

Eleven data elements reached consensus after round one ( Table 4). Twenty-six data elements did not reach consensus after round one ( Table 5); 66 data elements were excluded after round one ( Table 6).

**Table 4.** Data elements with consensus after round one.

Data element	Median score (IQR)	Agree percent
Unique Individual Identifier (i.e., ID)	5 (1)	81%
Age	5 (1)	92%
Sex	5 (1)	96%
Service Member is in Initial Military Training	5 (1)	96%
Body Part/Region	5 (1)	97%
Mechanism of Injury	5 (1)	90%
Presentation (i.e., sudden or gradual)	5 (1)	94%
Activity during injury	5 (1)	90%
Overuse Injuries	5 (1)	99%
Acute Injuries	5 (1)	99%
Traumatic Injuries	5 (1)	99%

IQR = Interquartile range.

Agree percent equates to a score of 4 or 5.

**Table 5.** Data elements with no consensus after round one.

Data element	Median score (IQR)	Percent agree
Height	4	67%
Weight	4	79%
Military Branch (e.g., Army, Navy, Air Force)	4	64%
Position, Duty or Occupational Specialty	4	76%
Years Working in the Military	4	64%
Terms of Service	4	58%
Previous Orthopaedic Surgery	4	73%
Body Part and/or Joint of Previous Orthopaedic Surgery	4	74%
How Long Ago Was Previous Orthopaedic Surgery	4	73%
What Type of Injury Was Past Injury (location, etc.)	4	78%
How Long Ago Was Previous Musculoskeletal Injury	4	72%
Percent Perceived Return to Function Following Previous Musculoskeletal Injury	4	69%
Tobacco Products Consumption	4	73%
Service Member is Attending Formal Military Training Course	4	69%
Service Member is in Special Operations	4	79%
Individual Exposure	4	76%
Exposure in Hours	4	52%
Exposure in Days	4	66%
Clinical Diagnosis of the Injury	4	71%
Days from Injury to Seeking Medical Care	4	64%
Days from Injury to Physical Therapy for Injury	4	59%
Duty Time Lost in Days from Injury	4.5	78%

**Table 6.** Data elements excluded after round 1.

Data element	Median score (IQR)	Percent agree
Waist Circumference	2 (1)	13%
Hip to Waist Ratio	2 (1)	6%
Body Fat Percentage	2.5 (1)	16%
Body Mass Index	2 (1)	7%
Race/Ethnicity	2 (1)	10%
Marital Status	1 (1)	6%
Physical Fitness Test Exemptions (e.g., “can do everything except running”)	2.5 (1)	24%
Previous Sport/Exercise Experience	2.5 (1)	18%
Historical Physical Fitness Test Scores	2.5 (1)	14%
Aerobic Fitness	2.5 (1)	27%
Rank	2 (1)	10%
Officer versus Ordinary Rank (i.e., Enlisted)	2 (1)	17%
Highest Level of Education Achieved	2 (1)	2%
Base or Post (i.e., where currently stationed)	2 (1)	9%
Unit	2 (1)	14%
Deployment History	2 (1)	15%
Number of Deployments	2 (1)	6%
Length of Most Recent Redeployment (months)	2 (1)	5%
Time Since Last Deployment (months/years)	2 (2)	6%
Menstrual Cycle/Amenorrhea	2.5 (1)	16%
Use of Oral Contraceptives	2.5 (1)	8%
Number of Pregnancies	2 (1)	8%
Number of Live Births	2 (2)	7%
Live Birth Complications History	2 (2)	5%
Time Since Last Live Birth	2 (1)	10%
Previous Time Loss from Injury (i.e., on profile, limited duty, light duty) Occurrences	2.5 (1)	32%
Previous Number of Days of Time Loss from Injury (i.e., on profile, limited duty, light duty)	2.5 (1)	33%
Treatment Interventions Used for Past Injury	2.5 (1)	17%
How Many Months Since Individual Passed Service Required Physical Fitness Test	2.5 (1)	16%
Joint Hypermobility	2 (1)	3%
Previously Seen a Mental Health Provider for a Mental Health/Behavioral Health Condition (and how long ago)	2.5 (1)	13%
Diagnosed Previous Sleep Disorders	2 (1)	9%
Previous History of Relative Energy Deficiency (RED-S)	2.5 (1)	15%
Previous History of Female Athlete Triad	2.5 (1)	19%
Self-Perceived Health	2 (1)	16%
Self-Perceived Fitness	2 (1)	16%
Recreational Physical Activity (e.g., per day, per week)	2.5 (1)	23%
Nutrition Habits	2.5 (1)	21%
Alcohol Consumption	2.5 (1)	16%
Contact and Collision Sport Involvement (and how long ago)?	2 (1)	13%
Childcare Responsibilities	2 (1)	6%
Social Support	2 (1)	13%
Emotional Stress	2.5 (1)	19%
Hours of Sleep	2.5 (2)	31%
Quality of Sleep	2.5 (1)	22%
Unit/Platoon Exposure	2.5 (1)	19%
Exposure in Minutes	2 (2)	6%
Number of Times/Activities Occurrences	2.5 (1)	22%
Work (exercise) Intensity	2.5 (1)	25%
Time Spent in Specific Activities of Varied Intensity	2.5 (1)	16%
Cumulative Loading Over Military Career	2.5 (1)	23%
Cumulative Loading Over Specified Period of Time (e.g., training exercise; deployment period)	2.5 (1)	31%
Laterality of Injury	2.5 (1)	20%
Amount of Pain from Injury	2 (1)	11%
Days from Initial Complaint Until Injury Became Time-Loss	2.5 (1)	13%
Time of Injury	2 (1)	11%
SNOMED CT Coding System	2 (1)	5%
Tissue	2.5 (2)	32%
Care-Seeking Injury	2.5 (2)	31%
Time Since They Previously Had That Injury If Prior History (months/years)	2.5 (1)	28%

IQR = Interquartile range.

Agree percent equates to a score of 4 or 5.

### Round two

In round two, 16 more data elements reached consensus ( Table 7); four data elements were combined with data elements that had already reached consensus ( Table 8). Six data elements did not reach consensus after round two ( Table 9).

**Table 7.** Included data elements after round two.

Data element	Median score (IQR)	Percent agree
Height	4 (1)	66%
Weight	4 (2)	74%
Military Branch	4 (1)	57%
Position Duty or Occupational Specialty	4 (2)	72%
Previous Orthopaedic Surgery	4 (2)	66%
Previous Musculoskeletal Injury	4 (2)	82%
Time Since Previous Musculoskeletal Injury	4 (2)	57%
Military Role (Combat, Support, Special Operations)	4 (1)	69%
Exposure in Days	4 (1)	60%
Clinical Diagnosis of Injury	4 (1)	77%
Days from Injury to Seeking Medical Care	4 (1)	52%
Duty Time Lost in Days from Injury	4 (1)	75%
Type of Injury (Primary, Subsequent, Recurrent, or Exacerbation Injury)	4 (1)	79%
Time Loss of Injury in Days	4 (1)	75%
Prior History of that Specific Injury	4 (1)	71%
Date of Injury	4 (1)	54%

IQR = Interquartile range.

Agree percent equates to a score of 4 or 5.

**Table 8.** Data elements combined with other elements (already with consensus) after round two.

Data element	Median score (IQR)	Percent agree	Combined with
Terms of Service	3 (2)	43%	Military Role
Exposure in Hours	3 (2)	48%	Exposure in Days
Days from Injury to Physical Therapy	3 (1)	42%	Days to Seeking Medical Attention
Surveillance Days Until Injury	3 (2)	45%	Days Until Injury

IQR = Interquartile range.

**Table 9.** No consensus after round two.

Data element	Median score (IQR)	Percent agree
Tobacco or Nicotine Use	3 (1)	45%
Terrain Where Injury Occurred	3 (1)	39%
Equipment Worn at the Time of Injury	3 (1)	40%
Years Working in Military	3 (2)	45%
Setting of training	3 (1)	46%
Formal Military Course	3 (1)	46%

IQR = Interquartile range.

Agree percent equates to a score of 4 or 5.

### Round three

Two more data elements reached consensus in round three. The other three data elements did not reach consensus ( Table 10).

**Table 10.** Round three.

Data element	Median score (IQR)	Percent agree
Tobacco or Nicotine Use	4 (3)	26%
Terrain Where Injury Occurred	3 (1)	22%
Equipment Worn at the Time of Injury	3 (1)	24%
Years Working in Military	3 (2)	21%
Setting of training	4 (2)	33%

IQR = Interquartile range.

Agree percent equates to a score of 4 or 5.

## Discussion

An integrated and scalable process for collecting and reporting MSKI data is necessary to successfully evaluate MSKI interventions in military populations. The methodology, supporting results, and decisions made during the ROMMIL development are provided in this report. The process included a literature synthesis, knowledge user survey, Delphi study, consensus meeting, and electronic consensus open meeting for further review. This process incorporated a broad range of supporting literature, relevant data, and direct input from knowledge users having wide-ranging MSKI-related experiences and expertise.

The steering committee included members with wide-ranging viewpoints and areas of expertise; military MSKI surveillance and research occur world-wide, encompassing varying levels of institutional support, environments, and cultures. Multiple knowledge user meetings were held across the research process. The closeness continuum, ^13^ identifying different types of experts, was used to inform and identify knowledge users for inclusion in the consensus process. The ROMMIL consensus statement was presented in person, on an open science platform, and through an open, freely accessible electronic link to mitigate or eliminate travel or institutional support barriers. Feedback was received through multiple interfaces, allowing for comprehensive input. Forms of feedback included verbal, free text, and quantitative scores, increasing the depth and richness of feedback.

### Potential limitations

While international input was emphasized, most knowledge user feedback came from participants living in English speaking countries (i.e. North America, Europe, former British Commonwealth countries), with the steering committee living in the United States and the United Kingdom. Military organizations with differing levels of institutional support may have differing (1) MSKI-related needs and (2) abilities to collect and report specific data elements. Further enquiry is needed to evaluate the ROMMIL checklist in these military populations. Only one conference was attended to help inform the original data elements list, as knowledge users from different backgrounds could attend other conferences across the globe, there may be selection bias limitations in the original proposed elements list. There was attrition in the Delphi survey, potentially decreasing the strength of recommendations. 

The ROMMIL steering committee screened data elements throughout the process, ensuring a manageable and feasible number of data elements was included for collection and reporting. Continual screening was necessary, given the wide-ranging (1) body of MSKI-related literature, (2) knowledge user suggestions, and (3) responses from the knowledge user survey and Delphi study. The steering committee might have selected data elements that differed from individual knowledge users’ selections. To mitigate this potential bias, the steering committee conducted multiple knowledge user meetings and encouraged maximal input/feedback from all members throughout the process. The primary report summarizing the ROMMIL consensus checklist for minimum data elements to collect and report when conducting injury surveillance research will be published separately.

## Conclusions

Achieving consensus with knowledge users representing global-wide military organizations is challenging. This paper details the methodology established to reach consensus, while enabling all participants to provide input (including dissenting opinions). The ROMMIL consensus provides a core minimum data elements checklist for military MSKI scientists to improve data harmonization and scalability efforts. These methods can be used as a resource to assist future consensus endeavors in similar populations.

## Ethics and consent

This project was determined to not be human subjects research by the Institutional Review Board at Wake Forest University, School of Medicine (# IRB00115873, determination made 1 July 2024). Individuals consented to be involved by participating in the meetings and voting process, a determination that was approved by the Institutional Review Board. All participants were made aware up front that the results and conclusions would be summarized into a consensus statement that would be published and shared with the greater scientific community. Data were anonymous and not linked to individual participants.

## Disclaimer

The view(s) expressed herein are those of the author(s) and do not necessarily reflect the official policy or position of the Uniformed Services University, the US Defense Health Agency, the US Department of Defense, the U.K. Department of Defense, nor the U.S. or U.K. Governments.

## Author information

The following authors are military service members (either past or present): JJF, BRH, JMM, DST, JMT, EW, SW, DVT, JJV, RBW, DIR.

## Data Availability

OSF: Supplementary Data for Project: Minimum Common Data Elements for surveillance and Reporting of Musculoskeletal Injuries in the MILitary (ROMMIL); DOI 10.17605/OSF.IO/2WQBR. ^15^ URL: https://osf.io/2wqbr/, ^15^ This project contains the following underlying data: This is a description of methodology and not a full report of the actual Delphi study, the results of which will be reported elsewhere. Delphi data
• DataDictionary.csv • Round1.csv • Round2.csv • Round3.csv *Data are available under the terms of the CC0 1.0 Universal* license applied. OSF: Supplementary Data for Project: Minimum Common Data Elements for surveillance and Reporting of Musculoskeletal Injuries in the MILitary (ROMMIL); DOI 10.17605/OSF.IO/2WQBR. ^15^ URL: https://osf.io/2wqbr/, ^15^ This project contains the following extended data: Initial questions
•
Appendix_Delphi_Initial_Tables.docx Recruitment email
• Delphi_Email.docx *Data are available under the terms of the CC0 1.0 Universal* license applied.

## References

[1] Rhon DI Molloy JM Monnier A : Much work remains to reach consensus on musculoskeletal injury risk in military service members: a systematic review with meta-analysis. *Eur. J. Sport Sci.* 2022;22(1):16–34. 10.1080/17461391.2021.1931464 33993835

[2] Mauntel TC Tenan MS Freedman BA : The military orthopedics tracking injuries and outcomes network: a solution for improving musculoskeletal care in the military health system. *Mil. Med.* 2022;187(3-4):e282–e289. 10.1093/milmed/usaa304 33242087

[3] Jones BH Canham-Chervak M Canada S : Medical surveillance of injuries in the US military: descriptive epidemiology and recommendations for improvement. *Am. J. Prev. Med.* 2010;38(1):S42–S60. 10.1016/j.amepre.2009.10.014 20117600

[4] Kush RD Warzel D Kush MA : FAIR data sharing: the roles of common data elements and harmonization. *J. Biomed. Inform.* 2020;107:103421. 10.1016/j.jbi.2020.103421 32407878

[5] McCann LJ Pilkington CA Huber AM : Development of a consensus core dataset in juvenile dermatomyositis for clinical use to inform research. *Ann. Rheum. Dis.* 2018;77(2):241–250. 10.1136/annrheumdis-2017-212141 29084729 PMC5816738

[6] Dworkin RH Turk DC Farrar JT : Core outcome measures for chronic pain clinical trials: IMMPACT recommendations. *Pain.* 2005;113(1):9–19. 10.1016/j.pain.2004.09.012 15621359

[7] Welch C Wilson D Sayer AA : Development of a UK core dataset for geriatric medicine research: a position statement and results from a Delphi consensus process. *BMC Geriatr.* 2023;23(1):168. 10.1186/s12877-023-03805-5 36959622 PMC10035483

[8] Bullock GS Dartt CE Ricker EA : Barriers and facilitators to implementation of musculoskeletal injury mitigation programmes for military service members around the world: a scoping review. *Inj. Prev.* 2023;29:461–473. 10.1136/ip-2023-044905 37620010 PMC10715562

[9] Ringdal KG Lossius HM Jones JM : Collecting core data in severely injured patients using a consensus trauma template: an international multicentre study. *Crit. Care.* 2011;15:1–11. 10.1186/cc10485 PMC3334788 21992236

[10] Leyland K Gates L Nevitt M : Harmonising measures of knee and hip osteoarthritis in population-based cohort studies: an international study. *Osteoarthr. Cartil.* 2018;26(7):872–879. 10.1016/j.joca.2018.01.024 29426005 PMC6010158

[11] Moher D Schulz KF Simera I : Guidance for developers of health research reporting guidelines. *PLoS Med.* 2010;7(2):e1000217. 10.1371/journal.pmed.1000217 20169112 PMC2821895

[12] Gattrell WT Logullo P Zuuren EJ van : ACCORD (ACcurate COnsensus Reporting Document): A reporting guideline for consensus methods in biomedicine developed via a modified Delphi. *medRxiv.* 2023. 2023.08.22.23294261. 10.1371/journal.pmed.1004326 PMC10805282 38261576

[13] Needham RD Loë RC de : The policy Delphi: purpose, structure, and application. *Canadian Geographer/Le Géographe Canadien.* 1990;34(2):133–142. 10.1111/j.1541-0064.1990.tb01258.x

[14] Donohoe HM Needham RD : Moving best practice forward: Delphi characteristics, advantages, potential problems, and solutions. *Int. J. Tour. Res.* 2009;11(5):415–437. 10.1002/jtr.709

[15] Supplementary Data for Project: Minimum Common Data Elements for surveillance and Reporting of Musculoskeletal Injuries in the MILitary (ROMMIL) Open Science Framework. 10.17605/OSF.IO/2WQBR Reference Source PMC11621606 39649840

[16] Landeta J : Current validity of the Delphi method in social sciences. *Technol. Forecast. Soc. Chang.* 2006;73(5):467–482. 10.1016/j.techfore.2005.09.002

[17] Nasa P Jain R Juneja D : Delphi methodology in healthcare research: How to decide its appropriateness. *World J Methodol.* Jul 20 2021;11(4):116–129. 10.5662/wjm.v11.i4.116 34322364 PMC8299905

