# Supplementary material for: Methodology used to develop the minimum common data elements for surveillance and Reporting of Musculoskeletal Injuries in the MILitary (ROMMIL) statement
Source: F1000Res. 2025 Apr 3;13:1044. Originally published 2024 Sep 11. [Version 2] doi: 10.12688/f1000research.152514.2 (PMC11621606; doi:10.12688/f1000research.152514.2)
Supplement: Peer review history

# Reviewer report: Reviewer response for version 2

**DOI:** 10.5256/f1000research.179630.r375434

**Published:** 2025-04-16

**Authors:** Oliver O'Sullivan

**Affiliations:** 1 University of Nottingham, Nottingham, England, UK

Dear authors, thank you for taking on board all the recommendations from the peer reviewers. I think the manuscript reads well, is clear and has an important message - as far as I am concerned, it is ready. Best wishes and well done.

Is the rationale for developing the new method (or application) clearly explained?

Yes

Is the description of the method technically sound?

Yes

Are the conclusions about the method and its performance adequately supported by the findings presented in the article?

Yes

If any results are presented, are all the source data underlying the results available to ensure full reproducibility?

Yes

Are sufficient details provided to allow replication of the method development and its use by others?

Yes

Reviewer Expertise:

Sports and exercise medicine, rehabilitation, military medicine

I confirm that I have read this submission and believe that I have an appropriate level of expertise to confirm that it is of an acceptable scientific standard.

# Reviewer report: Reviewer response for version 1

**DOI:** 10.5256/f1000research.167283.r339134

**Published:** 2024-12-05

**Authors:** Kenton R Kaufman

**Affiliations:** 1 Mayo Clinic, Rochester, USA

Abstract: 

- Please define a “mixed knowledge user”.
- Please describe how the “asynchronous mixed knowledge user meeting” was conducted, or is this a typographical error?  Please see comment in Methods section below.
- Recommend describing “civil servant practitioners” as a “civilian practitioner”. 

Introduction

- Please define a definition of “knowledge user”.

Methods

- When the core group pursued “a balance in expertise”, what expertise was being balanced?  What criteria were used to know when this goal had been achieved? 
- Stage 2: What was the military MSKI conference that was used to collect the convenience sample? How was this conference selected?  How was the stated goal to sample “global-wide subject matter experts” met?
- The methods state that a “synchronous mixed knowledge user meeting” was performed. The abstract states that an “asynchronous mixed knowledge user meeting” was conducted.  Is there a typographic error in the abstract?
- Recommend describing “civil servant practitioners” as a “civilian practitioner”. 

Results

- There was a >50% decrease in Administrative Leadership, Command Leadership, and Physical Training Instructor between Round 1 and Round 2 of the Delphi Study.  Was there any concern about this significant decrease in participation from these groups?

Discussion

- This study provides an important list of data elements to be used for surveillance and reporting of musculoskeletal injuries that occur during military training.  A weakness is that no data dictionary was created in this process and there is no indication that plans are in place to accomplish this task soon.

Is the rationale for developing the new method (or application) clearly explained?

Yes

Is the description of the method technically sound?

Yes

Are the conclusions about the method and its performance adequately supported by the findings presented in the article?

Yes

If any results are presented, are all the source data underlying the results available to ensure full reproducibility?

Yes

Are sufficient details provided to allow replication of the method development and its use by others?

Yes

Reviewer Expertise:

Musculoskeletal injury, Orthopedics, Registries, Outcomes Measurement

I confirm that I have read this submission and believe that I have an appropriate level of expertise to confirm that it is of an acceptable scientific standard, however I have significant reservations, as outlined above.

# Response

**Published:** 2025-03-18

**Authors:** Daniel Rhon

**Affiliations:** Department of Rehabilitation, Uniformed Services University, Bethesda, MD, USA

Thank you Dr. Kaufman for your valuable time and interest for reviewing this work and for the detailed feedback and recommendations for improving this manuscript. They are greatly appreciated. For clarity, we have copied your original remarks below and then provided a point-by-point response to each one.

 Thank you to the authors for their tremendous effort in addressing MSKI in military populations. The methods described for the development of the Minimum Data Elements for surveillance and Reporting of Musculoskeletal Injuries in the MILitary (ROMMIL) statement will be an important reference for future publications and work in this field.

 Overall, the article is clear, concise and well written. A few minor changes

*RESPONSE: Thank you for the positive sentiment towards our work.*

 Introduction:

 -Well written, intention and purpose are clearly described.

*RESPONSE:  Thank you.*

 Methods:

 -(Minor change): “ Military service members having sustained MSKI across their careers had subjective closeness. Clinicians treating service members for MSKI had mandated closeness. Researchers and scientific experts investigating military MSKI had objective closeness.” For individuals who qualified for more than 1 category, how were they assessed/allocated?

*RESPONSE: Thank you for this question. If a knowledge user was allocated to more than one group, they were included in both. We tried to create a balance between all three groups, and also have knowledge users in each group that were only in one group and were in multiple groups.*

 -(Minor change):Please include a brief discussion on what measures were taken to mitigate biases. 

*RESPONSE: Measures to reduce bias included having steering group members not being able participate in the small group conversations and only contributed to the full group discussions during the consensus meetings when invited. Steering group members were not allowed to initiate remarks. If invited to speak, steering group members were only allowed to provide clarification or guidance when appropriate.  An independent experienced external moderator lead all group discussions to reduce the risk of dominant personalities and also reduce steering group bias. Finally, all meetings were open for in person, video, and viewing at a later date, with an open period to comment, to allow for inclusion of voices that could not make the meetings. This has been clarified within the methods.*

 -(Minor change):Please describe dataset limitations within this section.

*RESPONSE: Data limitations have been added to the manuscript. “Only one conference was attended to help inform the original data elements list, as knowledge users from different backgrounds could attend other conferences across the globe, there may be selection bias limitations in the original proposed elements list. There was attrition in the Delphi survey, potentially decreasing the strength of recommendations.”*

 Results:

 -The results are presented clearly. No recommended changes.

*RESPONSE: Thank you for this comment.*

 Discussion/Conclusion:

 -(Minor change): Please provide a participant demographic breakdown, including profession. Given the challenges faced in achieving consensus, this information is relevant.

*RESPONSE: This is provided in Table 3, and also within the supplement.*

 -(Minor change): It would also be helpful if other limitations were explored that could have contributed to the consensus challenges.

*RESPONSE: Further limitations have been added, as discussed above. “Only one conference was attended to help inform the original data elements list, as knowledge users from different backgrounds could attend other conferences across the globe, there may be selection bias limitations in the original proposed elements list. There was attrition in the Delphi survey, potentially decreasing the strength of recommendations.”*

 -(Minor change): Please reframe the Discussion, or at least identify within the limitations, and amend the Conclusion to reflect this Delphi describes the United States and United Kingdom perspective, as indicated in the Methods section.

*RESPONSE: Further detail is included in the limitations.*

 Supplementary Files:

 -Thank you for including the Initial Tables file, it provides contexts and allows transparency around data bias.

*RESPONSE: You are welcome.*

# Reviewer report: Reviewer response for version 1

**DOI:** 10.5256/f1000research.167283.r339129

**Published:** 2024-11-27

**Authors:** Chris M. Edwards

**Affiliations:** 1 University of Sherbrooke, Sherbrooke, Québec, Canada

Thank you to the authors for their tremendous effort in addressing MSKI in military populations. The methods described for the development of the Minimum Data Elements for surveillance and Reporting of Musculoskeletal Injuries in the MILitary (ROMMIL) statement will be an important reference for future publications and work in this field. 

 Overall, the article is clear, concise and well written. A few minor changes 

 Introduction: 

 -Well written, intention and purpose are clearly described. 

 Methods: 

 -(Minor change): “ Military service members having sustained MSKI across their careers had subjective closeness. Clinicians treating service members for MSKI had mandated closeness. Researchers and scientific experts investigating military MSKI had objective closeness.” For individuals who qualified for more than 1 category, how were they assessed/allocated?

 -(Minor change):Please include a brief discussion on what measures were taken to mitigate biases.  

 -(Minor change):Please describe dataset limitations within this section. 

 Results:

 -The results are presented clearly. No recommended changes. 

 Discussion/Conclusion:

 -(Minor change): Please provide a participant demographic breakdown, including profession. Given the challenges faced in achieving consensus, this information is relevant. 

 -(Minor change): It would also be helpful if other limitations were explored that could have contributed to the consensus challenges. 

 -(Minor change): Please reframe the Discussion, or at least identify within the limitations, and amend the Conclusion to reflect this Delphi describes the United States and United Kingdom perspective, as indicated in the Methods section.

 Supplementary Files:

 -Thank you for including the Initial Tables file, it provides contexts and allows transparency around data bias.

Is the rationale for developing the new method (or application) clearly explained?

Yes

Is the description of the method technically sound?

Yes

Are the conclusions about the method and its performance adequately supported by the findings presented in the article?

Partly

If any results are presented, are all the source data underlying the results available to ensure full reproducibility?

Yes

Are sufficient details provided to allow replication of the method development and its use by others?

Yes

Reviewer Expertise:

Musculoskeletal injuries in military and emergency response personnel, female health in military and emergency response personnel, physical performance in military and emergency response personnel, mental health in military and emergency response personnel.

I confirm that I have read this submission and believe that I have an appropriate level of expertise to confirm that it is of an acceptable scientific standard.

# Response

**Published:** 2025-03-18

**Authors:** Daniel Rhon

**Affiliations:** Department of Rehabilitation, Uniformed Services University, Bethesda, MD, USA

Thank you Dr. Edwards for your valuable time and interest for reviewing this work and for the detailed feedback and recommendations for improving this manuscript. They are greatly appreciated. For clarity, we have copied your original remarks below and then provided a point-by-point response to each one.

 Abstract

- Please define a “mixed knowledge user”.

RESPONSE: A mixed knowledge user is denoting multiple groups in one meeting. As discussed in reviewer 1, the authors felt that this term should be deleted to provide clarity.

- Please describe how the “asynchronous mixed knowledge user meeting” was conducted, or is this a typographical error?  Please see comment in Methods section below.

RESPONSE: Mixed knowledge user was deleted from the manuscript. However, asynchronous knowledge user meeting is denoting meeting that have a mixture of live video conferencing and the ability of other knowledge users to watch the video later and give comments. This has been further clarified within the methods.

- Recommend describing “civil servant practitioners” as a “civilian practitioner”.

REPSONSE: this has been edited per the reviewer’s suggestion.

 Introduction

- Please define a definition of “knowledge user”.

RESPONSE: A knowledge user is a person who contributes as an active knowledge creator, who construct meaning in their own settings, and have input that is equal to those of the scientific community. Bullock GS, Fallowfield J, Fisher B, Whittaker JL, Gafari O, Bilzon JL. Prioritizing Knowledge User Engagement: Engaging Patients and the Public in Creating Enduring Musculoskeletal Rehabilitation Research. journal of orthopaedic & sports physical therapy. 2024 Dec;54(12):743-7.

 This has been further clarified within the introduction.

 Methods

- When the core group pursued “a balance in expertise”, what expertise was being balanced?  What criteria were used to know when this goal had been achieved?

RESPONSE: Expertise balance was focused on subjective, mandated and objective closeness. Subjective is lived experience in MSKI within the military, mandated is practioner/performance/physiology and methods in MSKI, and objective is specific research in military MSKI.

- Stage 2: What was the military MSKI conference that was used to collect the convenience sample? How was this conference selected?  How was the stated goal to sample “global-wide subject matter experts” met?

RESPONSE: The conference was Total Force Fitness MSK Health conference addressing human performance optimization and MSKI mitigation and treatment in service members. This was an international conference, across different professions in the military. This has been clarified within the methods.

  

- The methods state that a “synchronous mixed knowledge user meeting” was performed. The abstract states that an “asynchronous mixed knowledge user meeting” was conducted.  Is there a typographic error in the abstract?

RESPONSE: Thank you for bringing this to our attention. This was an asynchronous meeting. This has been edited.

  

- Recommend describing “civil servant practitioners” as a “civilian practitioner”.

RESPONSE: This has been edited per the reviewer’s suggestion.

 Results

- There was a >50% decrease in Administrative Leadership, Command Leadership, and Physical Training Instructor between Round 1 and Round 2 of the Delphi Study.  Was there any concern about this significant decrease in participation from these groups?

RESPONSE: It is true that there was attrition over time. Participants represent busy military leaders and clinicians, who travel often. We attempted to provide as large of a window as possible for responses to be submitted for those who could not attend; however, our hypothesis is that many individuals provided their input on the variables they felt of most importance in the very beginning. Those variables with consensus across the board did pass through to final stages.  Individuals were reminded multiple times that this was their opportunity to provide additional feedback, especially if they felt strongly about any remaining variable inclusion/exclusion.  It is possible that attrition was due to more ambivalence by some individuals towards the remaining variables.  This has also been highlighted in the limitations section.

 Discussion

- This study provides an important list of data elements to be used for surveillance and reporting of musculoskeletal injuries that occur during military training.  A weakness is that no data dictionary was created in this process and there is no indication that plans are in place to accomplish this task soon.

RESPONSE: Thank you for this comment.  The publication reporting the final publication does indeed provide guidance and examples to guide reporting of the recommended elements.

# Reviewer report: Reviewer response for version 1

**DOI:** 10.5256/f1000research.167283.r323555

**Published:** 2024-10-04

**Authors:** Oliver O'Sullivan

**Affiliations:** 1 University of Nottingham, Nottingham, England, UK

Dear Colleagues,

 Thank you for asking me to review Methodology used to develop the minimum common data elements for surveillance and Reporting of Musculoskeletal Injuries in the MILitary (ROMMIL) statement, which is a description of methodological approach taken to develop minimum data elements for Musculoskeletal injuries.

 This is an important area of work and much needed - you all should be applauding for grasping the nettle on this.

 I felt the manuscript was well written overall, but could be refined by addressing some of the points below. The most pressing is the use (and possible over-use) of the phrase Knowledge User - the definition seemed to shift and it wasn't clear by the end who you were actually talking about.

 However, the area of concern I have is with regard to the results of Delphi. They are partly presented in the results and in Table 4-10; however, you report that the full results will be published elsewhere. This seems to me to be a little bit odd - would it not be better to present them in full in this manuscript, or to remove the partial presentation from this manuscript to avoid double reporting/duplicate publications?

 I wish the authors Good luck with the next version

 Minor Revisions - 

 In your abstract, you capitalise Minimum Data Elements in the background, but then don't in the results/conclusions - could I suggest a consistent approach please?

 In abstract methods, you use a phrase 'civil servant practitioners' - do you think, given the international authorship and readership, that this term would translate? You could consider 'civilian' as a simpler alternative.

 The last sentence in the strategy section is exactly the same at the last sentence in the background section - suggest removing one of those.

 The fourth and fifth sentences in the Stage 1 paragraph appear to be saying 95% the same thing, but with a subtle difference. Do you require both? If so, suggest rephrasing for clarity.

 Your 'knowledge user' groups appear to change between the 'knowledge user involvement' and 'patient public involvement' sections. Either remove one of the elements in parenthesis if these phrases relate to the same people or please articulate the different knowledge user groups. In fact, you appear to change the knowledge user group composition in the PPI section (which is again different to above!) - please can you review this? If these are all different groups, composed of different individuals for a bespoke reason, please can you give the groups different names?

 Also, as an extension to above, please can you explain what you mean by 'mixed knowledge user'? Is that an amalgam of the three groups above, or something else?

 In stage 3 & 4, you refer to the steering committee. Are these the six individuals noted in the Strategy section? If so, please can you overtly make that point?

 The second sentence of Results sounds decidedly like an intro or methods sentence - please could you consider its inclusion?

 The second paragraph in the limitation appears repetitive - please could this be reviewed?

 You mention dissenting opinions in the conclusion, but I am struggling to see where these have been described or presented - please can you clarify this?

 In Table 3, the nationality for Round 2/3 'not disclosed' might be better in the title line, not in the same line as New Zealand

 Table 4 - 10 - please consider the implications of including these data if there is a plan for a further publication presenting said findings

Is the rationale for developing the new method (or application) clearly explained?

Yes

Is the description of the method technically sound?

Yes

Are the conclusions about the method and its performance adequately supported by the findings presented in the article?

Yes

If any results are presented, are all the source data underlying the results available to ensure full reproducibility?

Yes

Are sufficient details provided to allow replication of the method development and its use by others?

Yes

Reviewer Expertise:

Sports and exercise medicine, rehabilitation, military medicine

I confirm that I have read this submission and believe that I have an appropriate level of expertise to confirm that it is of an acceptable scientific standard, however I have significant reservations, as outlined above.

# Response

**Published:** 2025-03-18

**Authors:** Daniel Rhon

**Affiliations:** Department of Rehabilitation, Uniformed Services University, Bethesda, MD, USA

Thank you Dr. O’Sullivan for your valuable time and interest for reviewing this work and for the detailed feedback and recommendations for improving this manuscript. They are greatly appreciated. For clarity, we have copied your original remarks below and then provided a point-by-point response to each one.

 Dear Colleagues,

 Thank you for asking me to review Methodology used to develop the minimum common data elements for surveillance and Reporting of Musculoskeletal Injuries in the MILitary (ROMMIL) statement, which is a description of methodological approach taken to develop minimum data elements for Musculoskeletal injuries.

 This is an important area of work and much needed - you all should be applauding for grasping the nettle on this.

 I felt the manuscript was well written overall, but could be refined by addressing some of the points below. The most pressing is the use (and possible over-use) of the phrase Knowledge User - the definition seemed to shift and it wasn't clear by the end who you were actually talking about.

 However, the area of concern I have is with regard to the results of Delphi. They are partly presented in the results and in Table 4-10; however, you report that the full results will be published elsewhere. This seems to me to be a little bit odd - would it not be better to present them in full in this manuscript, or to remove the partial presentation from this manuscript to avoid double reporting/duplicate publications?

 I wish the authors good luck with the next version

**RESPONSE: **Thank you for these additional points. We have reviewed and considered them all carefully, to include addressing the “knowledge user” term.  We have also reviewed the tables (4-10) and wish to provide some further context. There is another publication that will report on the final checklist. However, the space constraints prohibit many of the details that expand on how we were able to reach the final consensus. This manuscript in F1000 provides much more granular details about the iterative steps taken to reach the final checklist. Therefore, we focused on methodology in this manuscript. If we provided the final checklist here, then there would also be copyright concerns to address when publishing it again in the final publication. The publication with the completed checklist refers back to this manuscript, to provide readers with further details about the process.

 Minor Revisions -

 In your abstract, you capitalise Minimum Data Elements in the background, but then don't in the results/conclusions - could I suggest a consistent approach please?

**RESPONSE:** Thank you for this comment. ‘Minimum Data Elements’ within the abstract background has been edited to lower case.

 In abstract methods, you use a phrase 'civil servant practitioners' - do you think, given the international authorship and readership, that this term would translate? You could consider 'civilian' as a simpler alternative.

**RESPONSE: **Yes, thank you. That is a great suggestion and we have made that change.

 The last sentence in the strategy section is exactly the same at the last sentence in the background section - suggest removing one of those.

**RESPONSE: **Thank you, we have removed the redundancy from the background section.

 The fourth and fifth sentences in the Stage 1 paragraph appear to be saying 95% the same thing, but with a subtle difference. Do you require both? If so, suggest rephrasing for clarity.

**RESPONSE: **Thank you, this has been consolidated to improve clarity.

 Your 'knowledge user' groups appear to change between the 'knowledge user involvement' and 'patient public involvement' sections. Either remove one of the elements in parenthesis if these phrases relate to the same people or please articulate the different knowledge user groups. In fact, you appear to change the knowledge user group composition in the PPI section (which is again different to above!) - please can you review this? If these are all different groups, composed of different individuals for a bespoke reason, please can you give the groups different names?

**RESPONSE: **Thank you for bringing this to our attention. The patient and public involvement section has been deleted, and the knowledge user group section has been updated for one paragraph.

 Also, as an extension to above, please can you explain what you mean by 'mixed knowledge user'? Is that an amalgam of the three groups above, or something else?

**RESPONSE:** Mixed knowledge user details knowledge users from across different groups. The term ‘mixed’ has been deleted from the manuscript as the authors felt this decreased the clarity in the methods.

 In stage 3 & 4, you refer to the steering committee. Are these the six individuals noted in the Strategy section? If so, please can you overtly make that point?

**RESPONSE:** Yes, the steering committee are the six individuals in the strategy section. This has been clarified.

 The second sentence of Results sounds decidedly like an intro or methods sentence - please could you consider its inclusion?

**RESPONSE: **This sentence has been moved to the methods section, under the statistical analyses subheading.

 The second paragraph in the limitation appears repetitive - please could this be reviewed?

**RESPONSE:** The second limitations sentence has been edited.

 You mention dissenting opinions in the conclusion, but I am struggling to see where these have been described or presented - please can you clarify this?

**RESPONSE: **The dissenting opinions will be published for full transparency in the supplementary appendix of the manuscript reporting the final checklist.

 In Table 3, the nationality for Round 2/3 'not disclosed' might be better in the title line, not in the same line as New Zealand

**RESPONSE: **This has been edited with a foot note stating that a blank cell denotes that the information was not disclosed.

 Table 4 - 10 - please consider the implications of including these data if there is a plan for a further publication presenting said findings

**RESPONSE:** Thank you for this discussion. As discussed in the first response, there is another publication that will report on the final checklist. However, the space constraints prohibit many of the details that expand on how we were able to reach the final consensus. This manuscript in F1000 provides much more granular details about the iterative steps taken to reach the final checklist. Therefore, we focused on methodology in this manuscript. If we provided the final checklist here, then there would also be copyright concerns to address when publishing it again in the final publication. The publication with the completed checklist will refer back to this manuscript, to provide readers with further details about the process.
